# Novel approach for the detection of intraperitoneal micrometastasis using an ovarian cancer mouse model

**DOI:** 10.1038/srep40989

**Published:** 2017-01-25

**Authors:** Ayesha B. Alvero, Dongin Kim, Eydis Lima, Natalia J. Sumi, Jung Seok Lee, Carlos Cardenas, Mary Pitruzzello, Dan-Arin Silasi, Natalia Buza, Tarek Fahmy, Gil Mor

**Affiliations:** 1 Division of Reproductive Sciences, Department of Obstetrics, Gynecology and Reproductive Sciences, Yale University School of Medicine, New Haven CT, USA; 2 Department of Biomedical Engineering, Yale University, New Haven, CT, USA; 3 Department of Pharmaceutical Sciences, Texas A&M HSC, College Station, TX, USA; 4 Department of Pathology, Yale University School of Medicine, New Haven CT, USA

## Abstract

Patients with epithelial ovarian cancer have the best overall survival when maximal surgical effort is accomplished. However, despite numerous technological advances, surgery still relies primarily on white-light reflectance and the surgeon’s vision. As such, micrometastases are usually missed and most patients clinically classified as a complete responder eventually recur and succumb to the disease. Our objective is to develop optical enhancers which can aid in the visualization of ovarian cancer micrometastasis. To this end we developed a nanoparticle (NP) platform, which is specifically targeted to the tumor microenvironment. Targeting is achieved by coating FDA-approved PLGA-PEG NP with the peptide sequence RGD, which binds with high affinity to αVβ3 integrins present in both the tumor-associated neovasculature and on the surface of ovarian cancer cells. Administration of the NP platform carrying fluorescent dyes to mice bearing intraperitoneal ovarian cancer allowed visualization of tumor-associated vasculature and its contrast against normal blood vessels. More importantly, we demonstrate the visualization of intraperitoneal ovarian cancer micrometastasis as small as 100 μm with optimal resolution. Finally, we demonstrate that the fluorescent dye cargo was able to penetrate intra-tumorally. Such modality could be used to allow microscopic surgical debulking to assure maximal surgical effort.

Ovarian carcinomas are treated by aggressive cytoreductive surgery followed by platinum- and taxane-based combination chemotherapy^1^. This standard of care results in an approximately 80% response rate; however, most patients eventually present with recurrent disease within the next five years^2^. It is in the recurrent setting that most patients succumb to the disease as co-presentation of carcinomatosis and chemoresistance limits the value of surgery and chemotherapy^3^. As such, prevention of recurrence has been an objective with the goal of improving patient survival.

Recurrence is thought to occur due to the presence of undetectable residual disease at the conclusion of first-line treatment^4^,^5^,^6^. Residual disease consists of microscopic chemoresistant cancer cells that survived chemotherapy and were missed at surgery. Approaches that have improved survival are those that minimize residual disease. Indeed it has been clearly demonstrated that ovarian cancer patients have the best overall survival when maximal surgical effort is accomplished^7^, meaning all tumors visible to the unaided eye of the surgeon have been resected. Current studies, however, show that over 50% of patients classified clinically as complete responders carry residual disease^8^,^9^.

Surgery relies on white-light reflectance and the surgeon’s vision. While identification of large metastases usually does not pose a challenge, micrometastases are impossible to distinguish intra-operatively. The use of fluorescent probes to aid in real-time surgical visualization is a rapidly expanding field^10^,^11^ but an even more promising approach is the encapsulation of fluorescent probes in nanoparticles (NPs)^12^,^13^.

NPs can encapsulate therapeutic or diagnostic agents and enhance their delivery to specific sites. Coating of NPs with polyethylene glycol (PEG) allow avoidance of the host’s reticuloendothelial system and confer the “stealth effect”^14^, which increases the chance of delivery to the targeted site. In addition to improved retention, a main advantage in the use of fluorescent NPs is that the NPs allow for conjugation of targeting molecules for improved delivery to target sites. In ovarian cancer the value of integrin αvβ3 in tumor targeting has been demonstrated. Integrin αvβ3 has been shown to be over- expressed in ovarian cancer cell lines and ovarian cancer tumors and the role of this integrin in ovarian cancer growth^15^,^16^ and metastasis formation^17^,^18^ has been described. Even more important, is the over-expression of integrin αvβ3 in ovarian cancer-associated neovasculature but minimal expression in normal quiescent blood vessels^19^,^20^. These findings provide a strong rationale to determine the value of αvβ3 in specifically targeting the ovarian cancer microenvironment.

Our objective is to develop specific tumor-targeting optical enhancers that can aid in visualization and delineation of intraperitoneal (i.p.) micrometastasis. Towards this goal we utilized a NP-based delivery system to target fluorescent probes to the ovarian cancer microenvironment via the tumor-associated neovasculature. Targeting is achieved by coating NP composed of FDA approved poly-lactic-co-glycolic acid (PLGA)^21^ and PEG with the peptide sequence arginine-glycine-aspartate (RGD). RGD binds with high affinity to αVβ3 integrins over-expressed in tumor-associated neovasculature as well as in ovarian cancer cells. Identification of micrometastasis is achieved by visualization of the abnormal vascularity and labeled micrometastasis as small as 100 μm. Such a platform may aid in the performance of microscopic tumor debulking with the goal of minimizing residual disease.

## Results

### Nanoparticle synthesis and characterization

**Figure 1:**
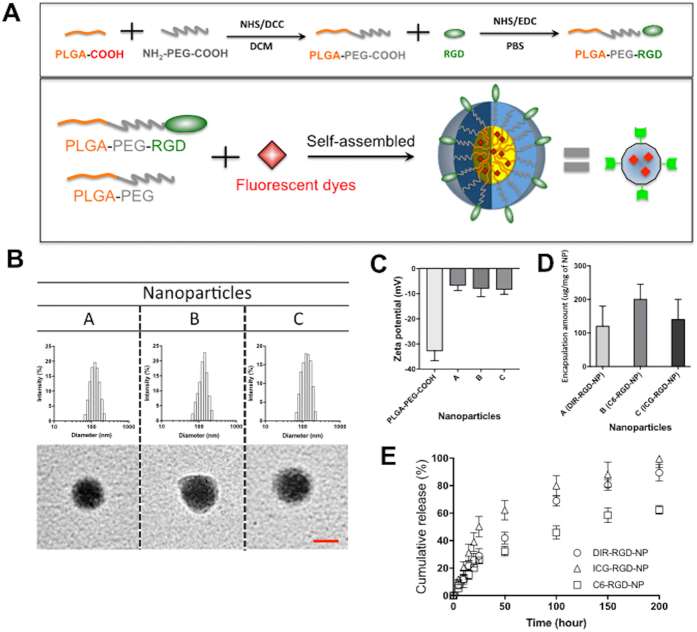
Fluorescent probes encapsulated RGD Nanoparticles. (**A**) Synthesis of RGD-NP composed of PLGA-PEG and its self-assembled NP. (**B**) NPs particle size was determined by DLC (upper panel) and TEM (down panel). (**C**) Surface charges of NP with and without RGD were compared. (**D**) Encapsulation amount of each dye within NP was also determined. (**E**) The release kinetic of each dye from NP was measured.

Our goal is to develop NPs that can deliver diagnostic agents to the ovarian cancer microenvironment and visualize micrometastasis and its associated neovasculature. With this objective we synthesized NPs composed of PLGA (5 K)-co-PEG (3.5 K) block copolymers. The carboxylic acid group at the end of PEG was used as the conjugation site for the RGD peptide and thus the RGD peptides are located on the surface of the NPs (Fig. 1a). We used three different fluorescent dyes - DIR, C6, and ICG (designated as DIR-RGD-NP; C6-RGD-NP; ICG-RGD-NP, respectively) and encapsulated them individually inside the PLGA hydrophobic core of the NPs (Fig. 1a). The sizes of these NPs range from 100 to 200 nm, which is confirmed by TEM images (Fig. 1b) and the surface charge is −5 to −7 mV. NPs not coated with RGD are −30 ± 5 mV since RGD conjugation takes up the negative charge of the carboxyl functional groups (Fig. 1c). The RGD conjugation ratio is 8 ± 5 (mol/mol %) against NP. The encapsulation amount for each fluorescent dye are as follows: 120 ± 60 μg/mg of NP for DIR; 200 ± 45 μg/mg of NP for C6; and 140 ± 60 μg/mg of NP for ICG (Fig. 1D). The release kinetic of DIR and ICG is faster than C6, which may be partly due to the higher hydrophobicity of C6 (Fig. 1e) such that the interaction between the hydrophobic core and C6 could delay its release.

### Specific staining of tumors and tumor-associated neovasculature with DIR-RGD-NP

**Figure 2:**
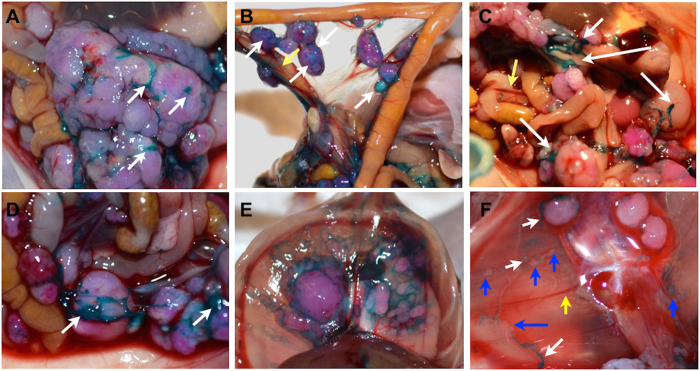
Staining and delineation of tumors and tumor-associated vasculature by DIR-RGD-NP. Upon establishment of tumors (ROI~40,000), mice were given four doses of DIR-RGD-NP (‘lower dose” indicated in Table 1) given every other day (n = 12). (**A**–**D**) Gross tumors appear distinctly stained compared to the intestines. Staining is specific to tumor-associated vasculature (white arrow), which can be easily contrasted to the normal vasculature (yellow arrow); (**E**,**F**) Tumors under the diaphragm are likewise stained; (**F**) Micrometastasis (blue arrow) is visible due to DIR-stained vessels (white arrow) and is contrasted against normal vessels (yellow arrow).

We initiated our *in vivo* studies using the DIR dye. DIR dye is a lipophilic, near infra-red dye with high tissue penetration and low autofluorescence^22^. DIR provides the advantage of being visible to the naked eye in addition to having a fluorescent signal. To determine the ability of DIR-RGD-NP to transport DIR to the ovarian tumor microenvironment we utilized the previously described ovarian cancer xenograft model established using mCherry-labeled ovarian cancer cells^23^,^24^. The mCherry fluorescence stably expressed by the cancer cells allows the prompt co-localization of the DIR signal with the established tumors. Supplementary Figure 1A–C shows the typical carcinomatosis formed by these cancer cells and the typical tumor growth progression quantified using mCherry ROI fluorescence as surrogate for tumor growth. Analysis of histological sections show morphologic features most consistent with a high-grade serous carcinoma, however, p53 immunostaining showed a wild-type staining pattern (Supplementary Figure 3). Supplementary Figure 1D shows the expression levels of αVβ3 integrins in the cancer cells utilized and the resulting i.p. tumor. Using this mouse model, 4 i.p. doses of DIR-RGD-NP were given every other day and mice were sacrificed 24 h after the last dose and analyzed post-mortem. Gross inspection of the abdomen showed intense staining (blue color) of tumor-associated vasculature in mice that received four doses of DIR-RGD-NP (corresponding to “lower dose” indicated in Table 1). With this dosing intense dark blue staining specifically localized to tumor-associated vasculature (white arrow) can be easily contrasted to the deep red normal vasculature (yellow arrow) (Fig. 2A–F). Tumor-associated neovasculature that were positively stained by the DIR-RGD-NP delivery system demonstrate the classical tortuous, disorganized, and blunt-ended morphology of tumor associated-vessels (Fig. 2F, white arrow) in contrast to the organized and linear morphology of normal vasculature (Fig. 2F, yellow arrow). Visualization of the stained tumor associated-vessels allowed identification of micrometastasis (Fig. 2F, blue arrow).

### Enhanced probe retention and co-localization *in vivo* when encapsulated in RGD-coated NP

**Figure 3:**
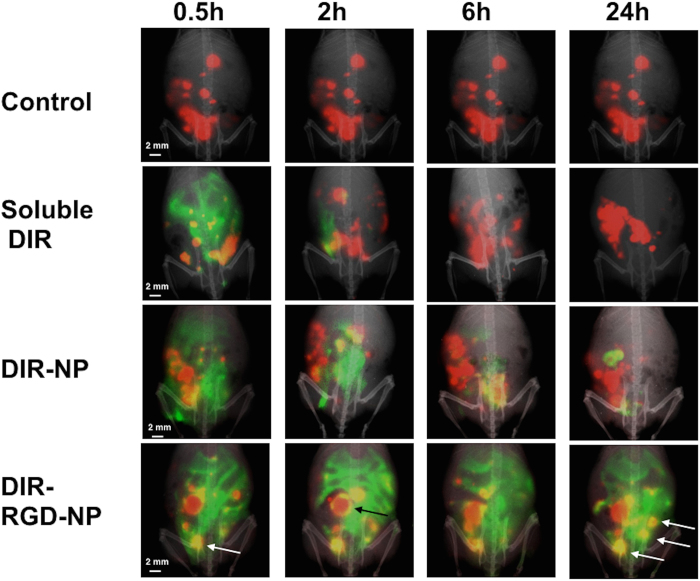
Enhanced retention and better colocalization *in vivo* with DIR-RGD-NP. Upon establishment of tumors (ROI~40,000), mice were given four doses of soluble DIR, DIR-NP, or DIR-RGD-NP given every other day. mCherry (red) and DIR (green) fluorescent images were obtained in live animals at designated time points 24 h after the 4^th^ dose. Images shown are merged images demonstrating the best colocalization of mCherry and DIR signals (yellow) in animals that received DIR-RGD-NP. A representative animal for each delivery system is shown; (n = 4 for soluble DIR and DIR-NP; n = 12 for DIR-RGD-NP).

To determine if both the NP and the RGD coating is required for the observed tumor staining in Fig. 2 (i.e. minimum component that is required to confer the best sensitivity and specificity), we tested the ability of soluble DIR, DIR encapsulated in naked NP (DIR-NP), and DIR-RGD-NP to co-localize with mCherr+ tumors. Fluorescent spectral imaging performed on live animals at designated time-points after the 4^th^ dose shows the transient retention of the DIR probe when it is administered in soluble form (Fig. 3). This agrees with previous data showing the rapid clearance of most fluorescent probe from the circulation^25^. A trivial improvement in the retention of DIR signal was observed when it was administered encapsulated in naked NP (DIR-NP) but the best retention was observed in mice that received DIR-RGD-NP (Fig. 3). Moreover, in these mice we observed the co-localization of the DIR and mCherry signals (yellow foci, white arrows bottom panel) in tumors equal or smaller than 2 mm from 30 mins after the last injection up to 24 h later. Co-localization in tumors bigger than 2 mm were however limited to the periphery of the tumors and is visualized as a yellow outline that surrounds the red tumor (Fig. 3, black arrow bottom panel). These results demonstrate that enhanced *in vivo* retention and better co-localization with big tumors are achieved when DIR is encapsulated in RGD-coated NP.

### Best tumor targeting is achieved with encapsulation in RGD-coated NP

**Figure 4:**
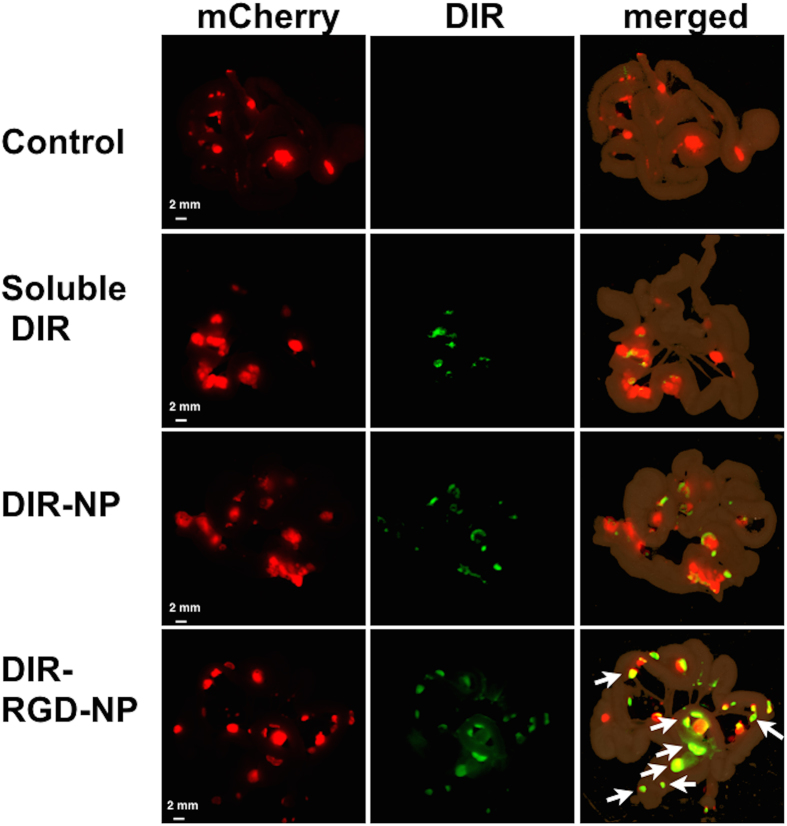
Staining of microtumors with DIR-RGD-NP. Fluorescence spectral images from dissected intestines and the attached mesentery. Images shown are from mCherry channel (red; left column) and DIR channel (green; middle column). The merged images (right column) demonstrate the best colocalization of mCherry and DIR signals (white arrow) in animals that received DIR-RGD-NP. A representative animal for each delivery system is shown; (n = 4 for soluble DIR and DIR-NP; n = 12 for DIR-RGD-NP).

Fluorescent spectral imaging and co-localization analysis were also performed *ex vivo* using dissected small intestines and the attached mesentery bearing multiple tumor implants (Fig. 4). Likewise, the best co-localization between DIR and mCherry signals were observed in mice that were administered DIR-RGD-NP. Pearson’s correlation coefficient was 0.5 to 0.64 in mice that were administered DIR-RGD-NP compared to 0.2 to 0.35 in mice that were administered DIR-NP (p = 0.008) (Supplementary Fig. 2). It should be noted that in mice that were administered DIR-RGD-NP, the co-localization was mostly with tumors equal or less than 2 mm (white arrows bottom panel), further supporting the platform’s ability to identify micrometastasis. Co-localization analysis could not be performed on mesenteries from control mice and mice that were administered soluble DIR due to lack of DIR signal.

**Figure 5:**
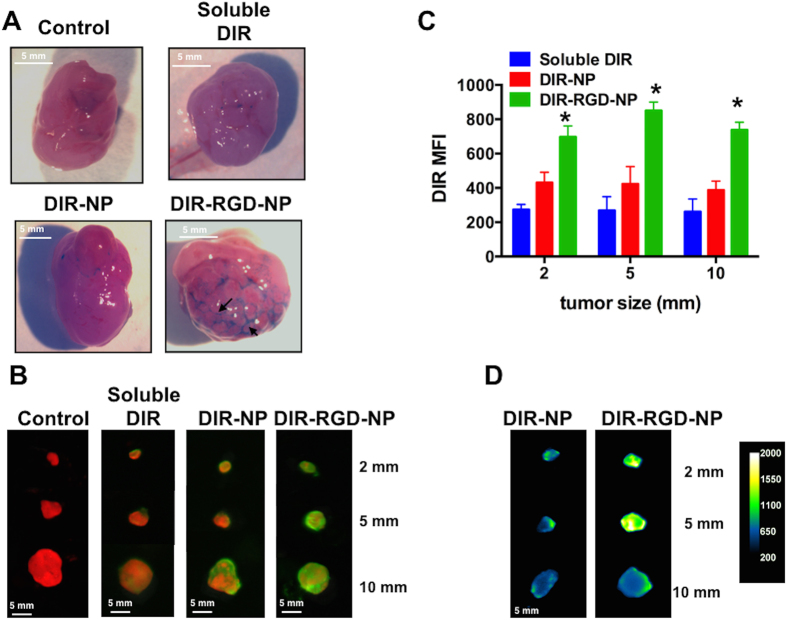
Delineation of tumor-associated vasculature with DIR-RGD-NP. (**A**) Delineation of tumor vascularity. White light image of tumors showing the outline of tumor-associated vasculature only when dye was administered in RGD-coated nanoparticles; (**B**) *Ex vivo* imaging and colocalization of mCherry and DIR signals in dissected tumors (from top: 2 mm, 5 mm, and 10 mm in size); (**C**) Quantification of DIR MFI, data shows mean ± SEM, *p < 0.002 compared to soluble DIR and p < 0.0237 compared to DIR-NP; (**D**) Quantification of DIR intensity and penetration in cross-sectioned tumors; a representative animal for each delivery system is shown in A, B, and D; (n = 4 for soluble DIR and DIR-NP; n = 12 for DIR-RGD-NP).

From each of the groups, we also analyzed representative i.p. tumors that range in size from 10 mm to 2 mm. Gross inspection of these tumors further show that only in those mice that received DIR-RGD-NP could we observe by eye the clear staining and delineation of the tortuous tumor-associated vasculature (Fig. 5A, black arrows). Fluorescent spectral imaging performed on these tumors recapitulated the observation that probe retention within the tumors is best achieved in mice that were administered DIR-RGD-NP (Fig. 5B). Indeed, quantification of DIR mean fluorescent intensity (MFI) demonstrates that tumors of various sizes obtained from mice administered DIR-RGD-NP demonstrate the highest MFI compared to tumors from mice that were administered DIR-NP and soluble DIR. Thus in tumors as small as 2 mm, DIR MFI in DIR-RGD-NP group is 696 compared to 430 (p = 0.02) and 274 (p = 0.002) in DIR-NP and soluble DIR groups, respectively (Fig. 5C). MFI in tumors from DIR-NP and soluble DIR groups was not significantly different (p = 0.289), suggesting the requirement for RGD for enhanced retention. The DIR signal can be seen to penetrate the core of tumors as small as 2 mm in size when DIR-RGD-NP is administered. As shown in Fig. 5D, quantification of DIR signal in dissected tumors that are cut cross-wise shows the best penetration of the DIR probe to the center of the tumor. Taken together our results demonstrate that the encapsulation of DIR in RGD-coated NP specifically targets the probe to tumor-associated vessels thus resulting in enhanced retention of the DIR signal in the tumor microenvironment and better co-localization with micrometastasis. This translates to higher fluorescent intensity within the tumors especially those that are less than 2 mm, which are the tumors that may be missed by the naked eye.

### Localization and delineation of micrometastasis

**Figure 6:**
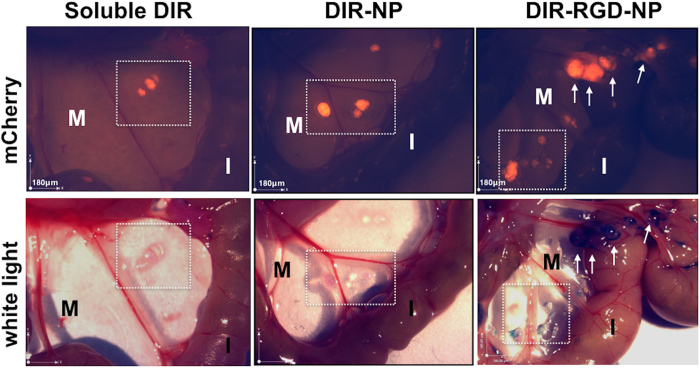
Identification of micrometastasis by white light. Stereoscopic images comparing specificity and sensitivity of the described nanoparticle platform (full data in Table 2). Squared area and white arrows point to micrometastasis. *M*, mesentery; *I*, intestines; a representative animal for each delivery system is shown; (n = 4 for soluble DIR and DIR-NP; n = 12 for DIR-RGD-NP).

To determine the ability of the delivery platform to locate and delineate micrometastasis, we analyzed the mice post-mortem under a stereoscopic microscope with resolution in the 100 μm range. Figure 6 shows representative images of mCherry and white light channels. Using this approach, we manually counted the following: (1) mCherry+ foci concurrently stained with DIR (true positive); (2) mCherry+ foci not stained with DIR (false negative); and (3) mCherry-negative foci stained with DIR (false positive). Table 2 summarizes the findings and demonstrate that we were able to identify 82% (±3%) of the micrometastasis in mice administered DIR-RGD-NP compared to 35% (±42%) and 0% in mice administered DIR-NP and soluble DIR, respectively. Correlation of mCherry signal and malignant status was further demonstrated by histopathological analysis (Supplementary Fig. 3).

### Sensitivity and specificity is maintained with one- time dosing

**Figure 7:**
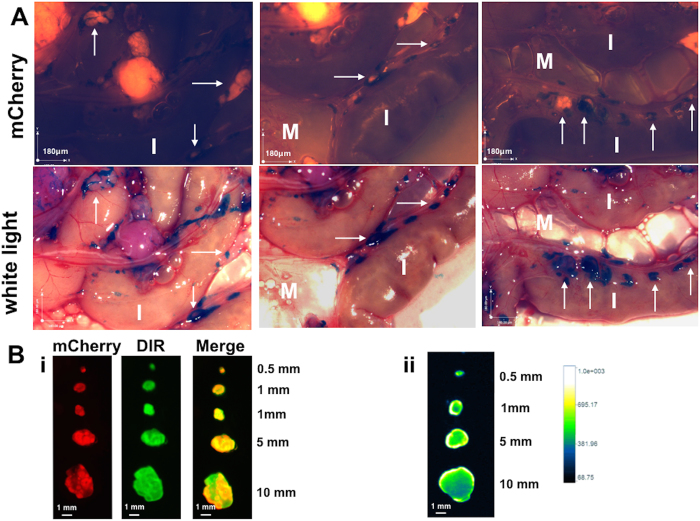
Specificity of detection is maintained with one time dosing of DIR-RGD-NP. (**A**) Upon establishment of tumors (ROI~40,000), mice were given a single dose of DIR-RGD-NP (“higher dose” indicated in Table 1; n = 4). Stereoscopic images show detection of micrometastasis by white light. White arrows point to micrometastasis. *M*, mesentery; *I*, instestines. (**B**) i, *Ex vivo* imaging and colocalization of mCherry and DIR signals in dissected tumors; ii, quantification of DIR intensity and penetration in cross-sectioned tumors.

To this point the experiments described above utilized 4 doses of the NP platform. For the DIR-RGD-NP group each dose corresponds to 300 μg of NP and 18 μg of DIR (designated as lower dose in Table 1). Our next objective was to determine the ability of a single dose to identify micrometastasis. For this we administered a single i.p. dose of DIR-RGD-NP at two different concentrations (lower dose and higher dose detailed in Table 1) and sacrificed the mice 24 h later. Similar to that observed with 4 doses, a single injection of DIR-RGD-NP at the higher dose was able to specifically stain micrometastasis. As such micrometastasis smaller than 100 μm can be easily visualized and delineated with optimal resolution (Fig. 7A, white arrows). Quantification of DIR MFI in dissected tumors 10 mm or less and further comparison with background DIR signal from intestines demonstrate signal to background ratio of 2 (±0.4) (Fig. 7Bi). Quantification of DIR signal in tumors that are cut cross-wise shows penetration of the DIR probe to the center of the tumor (Fig. 7Bii).

### Visualization of micrometastasis using Coumarin-6 and indocyanine green

**Figure 8:**
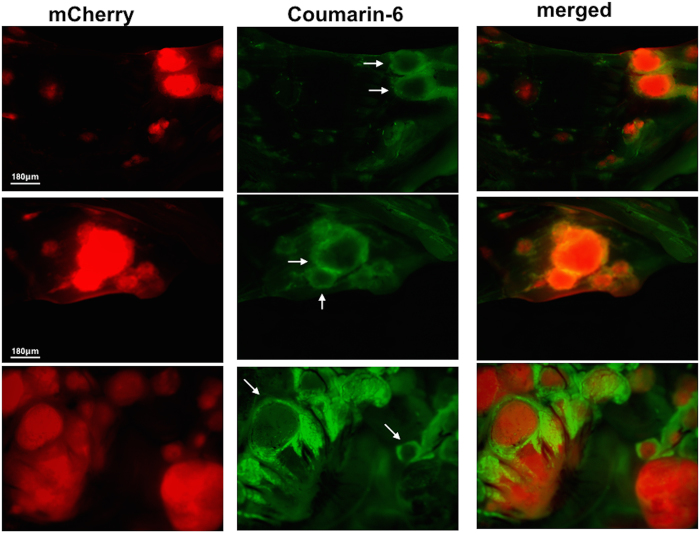
Biodistribution of C6-RGD-NP in the tumor microenvironment. Upon establishment of tumors (ROI~40,000), mice were given a single dose Cu6-RGD-NP and sacrificed after 2 h (n = 4). Fluorescence imaging demonstrates the spatial relationship between the DIR and mCherry signals; scale bar = 180 μm.

Our next set of experiment utilized RGD-coated PLGA NPs loaded with Coumarin-6 fluorescent probe (C6-RGD-NP). Coumarin-6 is a classical probe used to specifically track the biodistribution of nanoparticles^26^. Using the described model, mice bearing i.p. mCherry+ ovarian cancer were given a single i.p. injection of C6-RGD-NP. Post mortem analysis under a fluorescent dissection microscope was performed 2 h later and demonstrated spatial relationship between Coumarin-6 and mCherry signals. Coumarin-6 signal was observed as an outline that defines the border of mCherry+ tumors (Fig. 8, white arrows).

**Figure 9:**
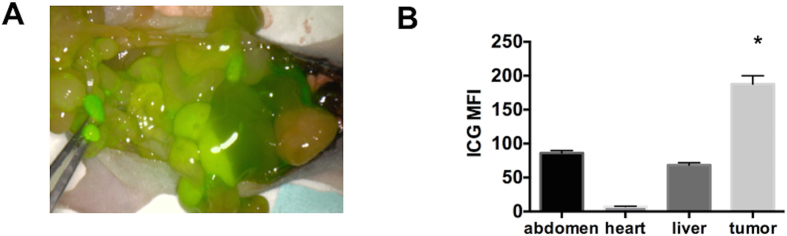
Visualization of micrometastasis using the ICG probe and the PINPOINT system. (**A**) Images from the PINPOINT system demonstrating the visualization of tumor-associated vasculature and micrometastasis (arrows); (**B**) quantification of ICG intensity showing most intense staining in tumors less than 5 mm compared to the rest of the abdomen, data shows mean ± SEM,*p < 0.0001 compared to abdomen, heart, and liver (n = 4).

Finally, we tested the FDA approved fluorescent dye indocyanine green (ICG)^27^ and utilized the PINPOINT endoscopic fluorescence imaging system (Fig. 9A). Tumors less than 2 mm were dissected and their fluorescent intensity compared to that of surrounding normal tissue including the heart and liver. Whereas tumors that are readily visible to the naked eye demonstrate similar fluorescence levels as the surrounding tissues, small micrometastasis that would otherwise be missed demonstrate significantly higher fluorescence compared to the surrounding abdomen, heart, and liver (p < 0.001, Fig. 9B). Thus taken together our results demonstrate that independent of the fluorescent probe, DIR-RGD-NP provides a sensitive and specific targeting platform for the localization of intra-peritoneal micrometastasis.

## Discussion

We report the characterization of a NP platform that is able to identify and delineate micrometastasis in an intraperitoneal xenograft model of ovarian cancer. Our results demonstrate that encapsulating fluorescent dyes in PLGA NPs and coating of NPs with RGD allows for specific targeting and enhanced retention to the tumor microenvironment. Consequently, the encapsulated probe is delivered and able to specifically delineate intra-peritoneal micrometastasis as small as 100 μm.

Clinical studies have previously demonstrated that soluble probes are rapidly cleared and are not optimally delivered to the tumor microenvironment. In ovarian cancer patients, studies have shown that the use of soluble ICG dye intra-operatively leads to a false positive rate of 62%^28^. Indeed it has been suggested that modalities that aim to improve surgical detection of residual disease or micrometastaisis need to be tumor-specific. Asanuma *et al*. recently reported the preclinical potential of β-galactosidase-targeting probes in the detection of micrometastasis in ovarian cancer xenografts^29^. In this current study, we demonstrate a better level of detection using materials that are already FDA approved and currently used in the clinic. We demonstrate that we are able to visualize with optimal resolution micrometastasis as small as 100 μm.

A major problem in tumor cell targeting is the lack of specific cellular markers that are unique to the targeted tumor. There are a very limited number of proteins that are expressed only by the cancer cells and this number is even more restricted for cell surface proteins. On the other hand, there is an expansive set of studies that describe the uniqueness of tumor-associated neovasculature, not only in terms of its morphology, but also in terms of the cell surface markers that are expressed by the newly formed endothelial cells. Integrin αvβ3 is either absent or weakly expressed in normal endothelial cells but is highly expressed in new and activated endothelial cells especially in tumor-associated endothelial cells^30^. Indeed, it has been reported that a considerable number of colon, pancreas, lung, and breast carcinoma lesions have vessels that express high levels of αvβ3^31^,^32^,^33^,^34^. In addition, αvβ3 has also been demonstrated to be highly expressed in cancer cells including ovarian^18^. The RGD peptide has been demonstrated to function as a specific targeting ligand for αvβ3 integrins and has been shown to be an optimal targeting agent^35^. We demonstrate in this study that indeed, the RGD-coated NP platform can specifically stain tumor-associated neovasculature and as a result can allow the localization and delineation of adjacent micrometastasis. Although the use of RGD-coated NPs have been previously described especially as a platform for drug delivery^30^ this is the first demonstration of its value in identifying and delineating micrometastasis. We demonstrate that we are able to visualize, with optimal resolution, micrometastasis as small as 100 μM.

We show that RGD-coated NPs specifically bind to the tumor-associated vessels and not normal vessels. Independent of the fluorescent probe used the NP system was able to stain tumor-associated vessels and demonstrate its tortuous morphology. Based on the distribution of the fluorescence, we speculate that the signal observed in the tumor microenvironment is mainly coming from the NP bound to tumor endothelial cells. However, we can not disregard the possibility of direct binding to cancer cells since ovarian cancer cells also express αvβ3 integrins^16^,^30^,^36^,^37^.

Once the DIR-RGD-NP binds to tumor-associated vessels the subsequent release of the dye allows diffusion into and therefore fluorescent labeling of the cancer cells. This especially occurs in smaller tumors, which provides an assurance for the identification of micrometastasis that can not be detected by the naked eye.

Both the PLGA NP and the ICG fluorescent probe are re FDA approved and their safety has been well established. PLGA is clinically used in a variety of biomedical applications, such as, grafts, sutures, implants, prosthetic devices, and surgical sealant films. PLGAs are also being used as microparticles and nanoparticles for drug delivery. Therefore, the platform described in this study is composed of well-established and FDA-approved components, which is a major advantage in its route for diagnostic applications.

The proposed approach may aid in improved microscopic surgical debulking and consequently minimize residual disease. In addition, such an approach may improve the visualization of tumor margins and hence reduce inadvertent injury to vital structures. Such a modality can assure the best surgical effort and may have significant impact on patient survival.

## Methods

### Nanoparticle synthesis

The procedure for the conjugation of acid-terminated PLGA (MW: 5 K) and amine-terminated PEG (MW: 3.5 K) was modified based on previously reported methods^38^. Acid-terminated PLGA (500 mg) and a 10-fold excess of N-hydroxysuccinimide (NHS) and N,N′-dicyclohexyl carbodiimide (DCC) were dissolved in 10 mL of anhydrous dichloromethane (DCM). After stirring at room temperature for 4 hours, the solution was filtered through a PTFE filter to remove the precipitate. The NHS-activated PLGA was obtained by precipitation in cold ethyl ether. After drying under a vacuum, NHS-activated PLGA was dissolved in anhydrous DCM with an equivalent molar ratio of NH_2_-PEG-COOH and the solution was stirred at room temperature. The conjugate was precipitated in cold ethyl ether and dried under a vacuum, yielding a precipitate above 90%. The RGD peptide was conjugated with the carboxylic group of PLGA-PEG-COOH using NHS and 1-ethyl-3-(3-dimethylaminopropyl)carbodiimide (EDC). Using this block co-polymer a fluorescent dye was encapsulated into the nanoparticles via dialysis. Specifically, the dye and polymer were dissolved in dimethyl sulfoxide (DMSO) and the solution was transferred to a dialysis membrane (MWCO 100,000). The dialysis was performed for 24 hours against DI water. After this time, the aqueous particle solution was centrifuged and sonicated to concentrate the particles.

### Nanoparticle characterization

The size of the nanoparticles was determined by dynamic light scattering (DLS) using a Zetasizer (Malvern, MA). The sample concentration was maintained at 0.5 mg/mL. The amount of dye encapsulation was determined from its absorbance measurement upon dissolving 10 mL of dye NPs into 990 mL of DMSO, which releases dye into the DMSO solution. Fluorescent dyes used in this study were: (1) DIR (1,1′-dioctadecyl-3,3,3′,3′-Tetramethylindotricarbocyanine Iodine, AAT Bioquest, CA); (2) Coumarin-6 (C6) (3-2(Benzothiazolyl)-7-(diethylamino)cuomarin, Sigma-Aldrich, MO); and (3) Inodcyanine green (ICG) (Akorn, Inc., Lake Forest, IL) and their absorbance wavelengths are 748, 443, and 800 nm, respectively. Absorbance was then measured at each wavelength of dye. The encapsulated dye concentration was calculated using a pre-measured calibration curve of dye absorbance according to its titrated concentration,. To determine the dye release profile one milliliter of PBS-dye NPs was prepared in a 1.5 mL microcentrifuge tube with moderate shaking. At each time point, the tube was centrifuged to pellet the NPs and the supernatant was collected. The supernatant was diluted 1:100 with DMSO, and its absorbance was measured at each dye wavelength. Transmission electron microscopy (Tecnai Osiris 200 kV TEM, FEI, Hillsboro, USA) was performed to elucidate the morphology of the NPs. A dispersion of the NPs (2 μL) was placed on a 400-mesh carbon grid followed by staining with a phosphotungstic acid solution (1 wt.%). The excess solution was removed and the sample was dried at room temperature for the measurement.

### Cell lines and culture conditions

The mCherry–labeled ovarian cancer cell line OCSC1-F2 (mCherry+ OCSC1-F2) was generated and propagated as previously described^23^,^24^. These cells were isolated from ascites from a patient diagnosed with a low-grade serous carcinoma arising in a serous borderline tumor. This cell line is not in the list of commonly misidentified cell line (www.iclac.org). mCherry fluorescent protein was stably introduced using lentivirus. Mycoplasma testing is performed prior to each experiment.

### Establishment of ovarian cancer xenograft and administration of fluorescent probes

The Yale University Institutional Animal Care and Use Committee approved all of the *in vivo* studies described and all methods were performed in accordance with the relevant guidelines. Intra-peritoneal tumors were established by intra-peritoneal (i.p.) injection of 4 × 10^6^ mCherry+ OCSC1-F2 cells in 6–8 weeks female athymic nude mice. I.p. tumor growth was monitored q3d by live imaging using *In Vivo* FX PRO (Bruker Corp., Billerica, MA) and quantified using the mCherry fluorescence region of interest (ROI) area as a surrogate for tumor growth. Animals were randomized into groups and the described probes were administered i.p. when the mCherry ROI area was between the range of 40,000 and 50,000. DIR dye in the following delivery systems were administered: (1) soluble; (2) encapsulated in naked NP; (3) encapsulated in RGD-coated NP. C6 and ICG were also encapsulated in RGD-coated NP and administered similarly. The concentration of nanoparticle and concentration of each dye are listed in Table 1. All animals were included in the analysis.

### Fluorescence spectral imaging, quantification, and colocalization analysis

Live *in vivo* x-ray and fluorescence images were obtained using *In Vivo* MS FX PRO (Bruker, Inc., Billerica, MA) in mice that were administered 2% isoflurane. Quantitative analysis of the optical signal capture was performed using Bruker MI Software. Fluorescence filters were as follows: (1) mCherry excitation = 550 and emission = 635; (2) DIR excitation = 760 and emission = 700; and (3) Coumarin 6 excitation = 450 and emission = 700. Fluorescence intensity for each of the probes was determined and reported as mean fluorescent intensity (MFI). Images were overlaid and analyzed for colocalization (Pearsons’s correlation coefficient) using ImageJ software (v 2.0). For all analysis, the investigators were not blinded to the group allocation.

### PINPOINT endoscopic system

The endoscopic PINPOINT system was operated at 5 cm working distance from the animal surface, which allows the animal’s entire abdomen to be viewed in a single field of view. Semiquantitative comparison of the distribution of ICG NPs was based on fluorescence intensity measured from different areas of the animal abdomen using ImageJ software.

### Statistical analysis

Data were graphed and analyzed using GraphPad Prism. Significance was calculated using one-way ANOVA or two-way ANOVA with Tukey’s correction for multiple comparisons, and p < 0.05 was considered significant.

## Additional Information

**How to cite this article**: Alvero, A. B. *et al*. Novel approach for the detection of intraperitoneal micrometastasis using an ovarian cancer mouse model. *Sci. Rep.* **7**, 40989; doi: 10.1038/srep40989 (2017).

**Publisher's note:** Springer Nature remains neutral with regard to jurisdictional claims in published maps and institutional affiliations.

## Supplementary Material

Supplementary Information

## Figures and Tables

**Table 1:** Nanoparticle and dye concentration per dose.

	Nanoparticle (μg/dose)	Dye (μg/dose)
Deep infrared (Dir): lower dose	300 μg	18 μg
Deep infrared (Dir): higher dose	600 μg	36 μg
Coumarin-6 (C6)	300 μg	30 μg
Indocyanine green (ICG)	300 μg	21 μg

**Table 2:** Manual counts of detected (mCherry+/DIR+) and missed (mCherry+/DIR-) micrometastasis.

group	% detected	SD
Soluble DIR	0	
DIR-NP	34.68	42.86
DIR-RGD-NP	81.5	3.7
**group**	**% missed**	**SD**
Soluble DIR	100	
DIR-NP	65.32	42.86
DIR-RGD-NP	18.50	3.7
