# Supplementary material for: Novel approach for the detection of intraperitoneal micrometastasis using an ovarian cancer mouse model
Source: Sci Rep. 2017 Jan 25;7:40989. doi: 10.1038/srep40989 (PMC5264387; doi:10.1038/srep40989)

Novel approach for the detection of intraperitoneal micrometastasis using an ovarian cancer mouse model

Ayesha B. Alvero, Dongin Kim, Eydis Lima, Natalia J. Sumi, Jung Seok Lee, Carlos Cardenas, Mary Pitruzzello, Dan-Arin Silasi, Natalia Buza, Tarek Famhy and Gil Mor

Supplementary Figure 1. mCherry-labeled ovarian cancer xenograft model. mCherry+ ovarian cancer cells were injected i.p. **(A)** Gross morphology of resulting carcinomastosis; **(B)** typical growth progression; **(C)** tumor kinetics based on mCherry fluorescence area; and **(D)** western blot analysis for integrin αv and integrin β3 in ovarian cancer cells and the resulting i.p. tumors.

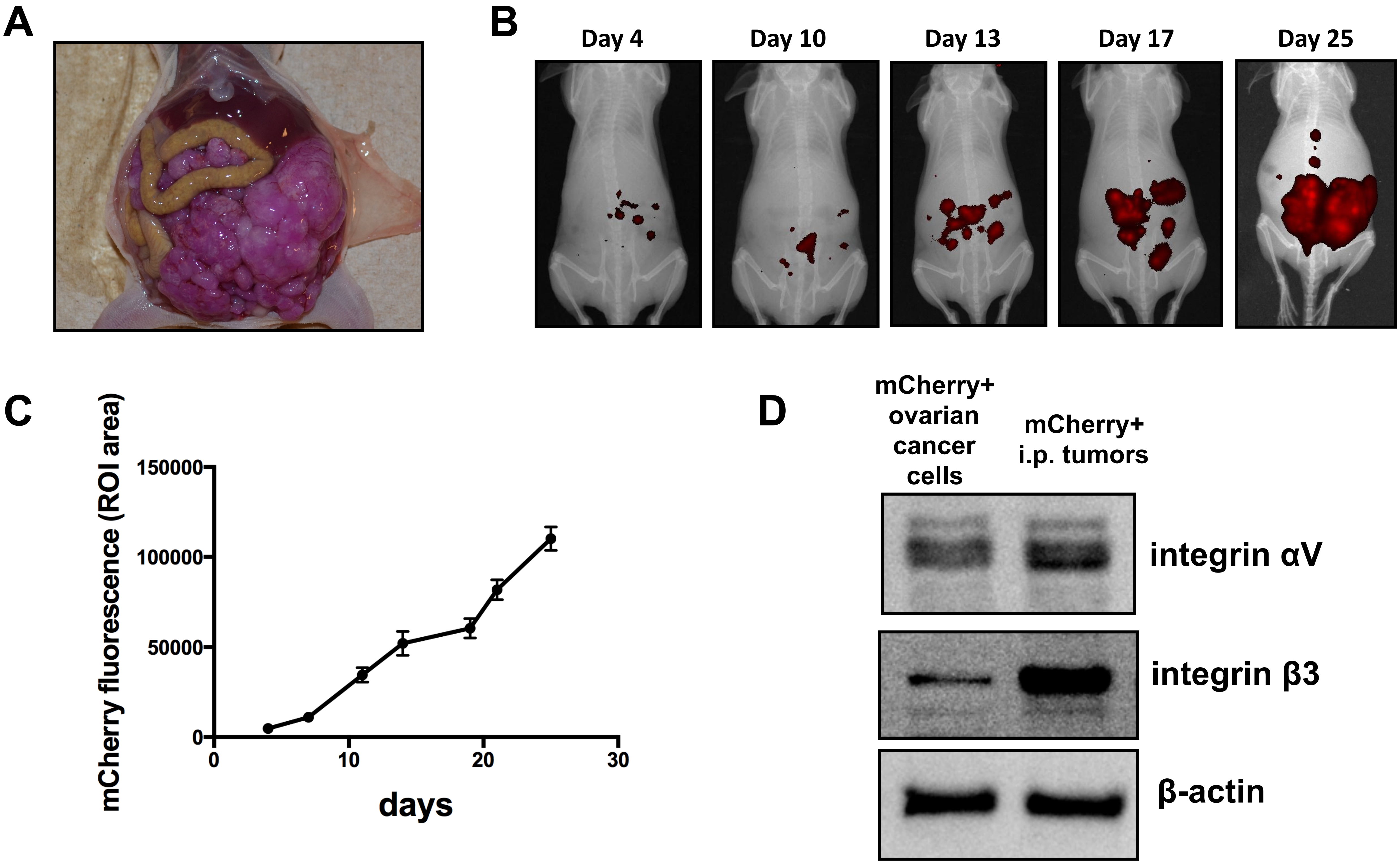

Supplementary Figure 2. Pearson's correlation coefficient obtained from DIR and mCherry signals from dissected intestines in Figure 4, * p = 0.008.

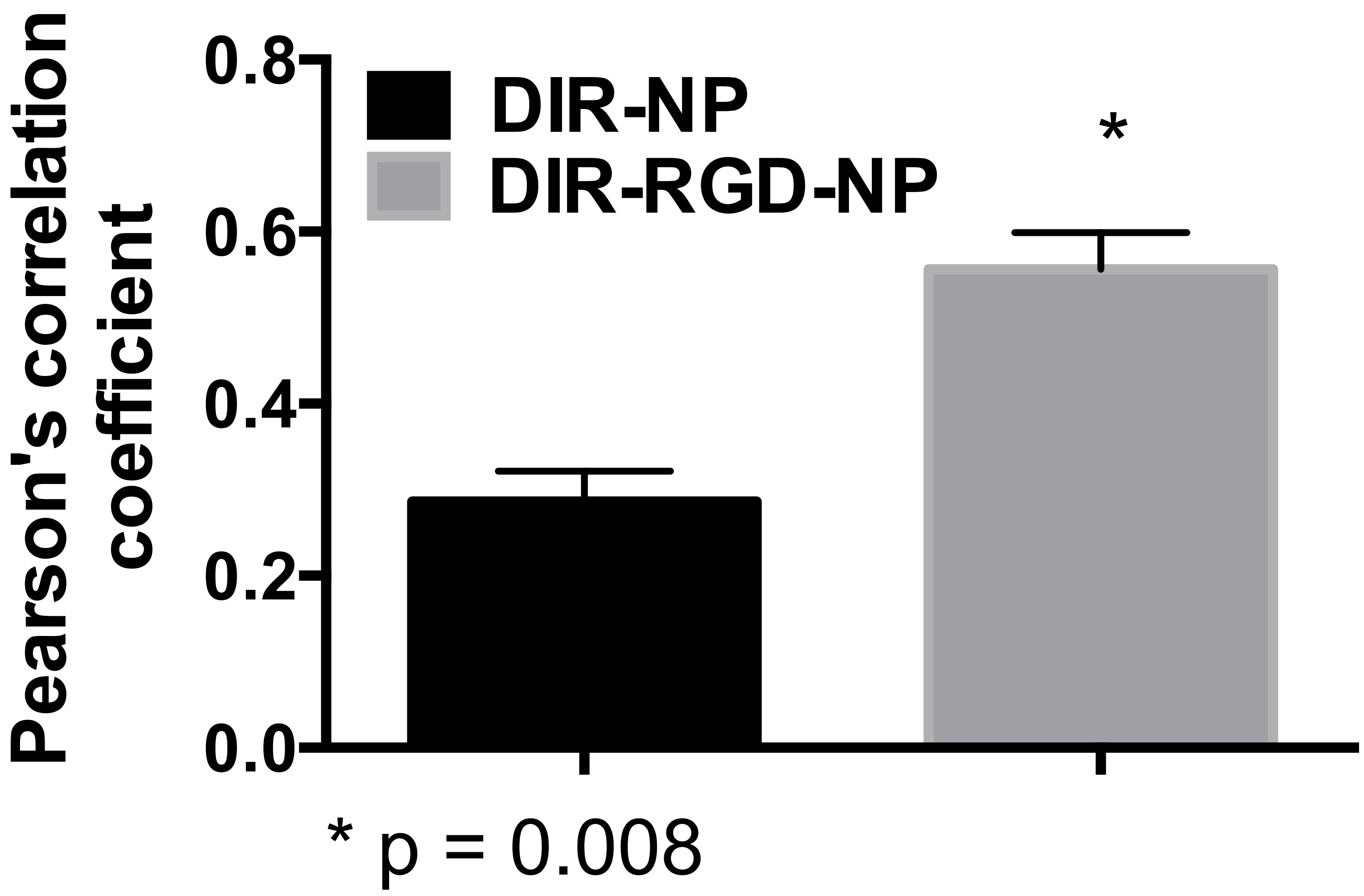

Novel approach for the detection of intraperitoneal micrometastasis using an ovarian cancer mouse model

Ayesha B. Alvero, Dongin Kim, Eydis Lima, Natalia J. Sumi, Jung Seok Lee, Carlos Cardenas, Mary Pitruzzello, Dan-Arin Silasi, Natalia Buza, Tarek Famhy and Gil Mor

Supplementary Figure 3. Histopathology results conclusively demonstrating that DIR+ lesions are malignant. **(A-B)** The tumor cells form solid nests and sheets with areas of necrosis. The nuclei are large, but relatively uniform; the nuclear to cytoplasmic ratio is high and there is brisk mitotic activity (> 50/ 10 HPF). **(C)** The tumor cells show high nuclear to cytoplasmic ratio and numerous mitotic figures (H&E stain, 400x original magnification). **(D)** p53 immunostaining shows a normal, wild type staining pattern: mild to moderate nuclear reactivity in approximately 10-20% of tumor cells.

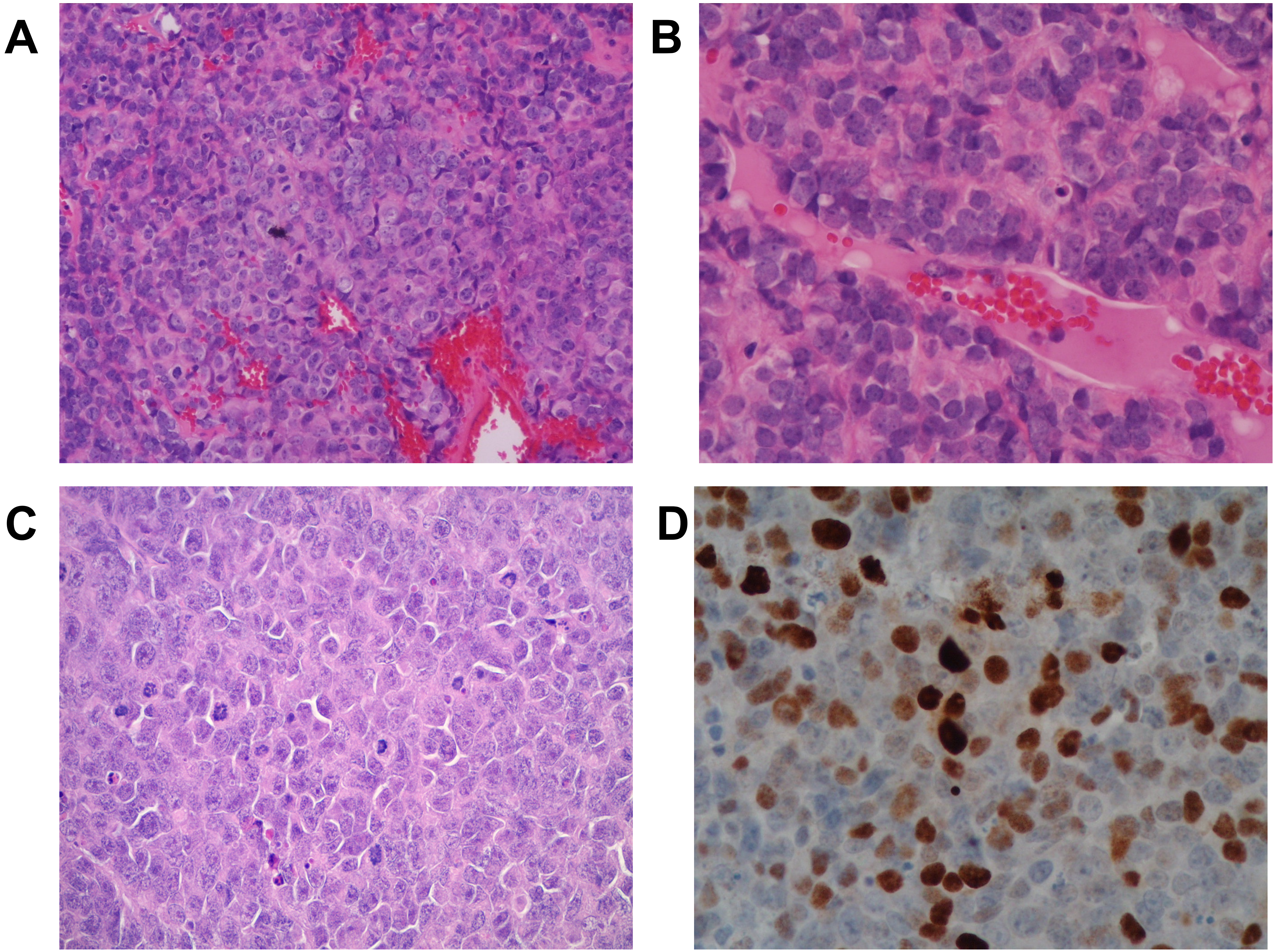

Supplement: Supplementary Information [file srep40989-s1.pdf]
